# *“So that’s why I found PrEP to be safest way to protect yourself”*: exploring IPV experiences and impact on HIV prevention among pregnant and postpartum women in Cape Town, South Africa

**DOI:** 10.1186/s12889-024-17871-w

**Published:** 2024-02-15

**Authors:** Amanda P. Miller, Sarah Schoetz Dean, Lara Court, Rufaro Mvududu, Nyiko Mashele, Nafisa J. Wara, Landon Myer, Steven Shoptaw, Dvora L. Joseph Davey

**Affiliations:** 1 Division of Epidemiology and Biostatistics, School of Public Health, San Diego State University, San Diego, CA USA; 2 Division of Infectious Diseases, David Geffen School of Medicine, University of California, Los Angeles, Los Angeles, CA USA; 3 Department of Epidemiology, Fielding School of Public Health, University of California Los Angeles, Los Angeles, CA USA; 4 Division of Socio-behavioural Sciences, School of Public Health and Family Medicine, University of Cape Town, Cape Town, South Africa; 5 Division of Epidemiology and Biostatistics, School of Public Health and Family Medicine, University of Cape Town, Cape Town, South Africa; 6 David Geffen School of Medicine, University of California, Los Angeles, Los Angeles, CA USA

**Keywords:** Intimate partner violence, HIV, Alcohol use, South Africa, Pregnancy, PrEP

## Abstract

Intimate partner violence (IPV) occurs at alarmingly high rates towards pregnant women in South Africa. Experiences of emotional, physical, and sexual IPV in pregnancy can adversely impact the health and safety of mother and fetus. Furthermore, IPV is associated with increased risk of HIV, exacerbating the public health impact of violence among pregnant women in this HIV endemic setting. In-depth understanding of cultural and contextual drivers of experiences of IPV is a critical precursor to development of interventions effectively addressing this issue among pregnant women in South Africa. The present study examines factors contributing to IPV among pregnant women to identify potential points of intervention. We conducted twenty in-depth interviews with postpartum women who used oral pre-exposure prophylaxis (PrEP) in pregnancy and reported recent experiences of IPV and/or ongoing alcohol use in a township near Cape Town, South Africa that experiences a heavy burden of both HIV and IPV. Interpretive thematic analysis was used. Several patterns of IPV during pregnancy were identified and violence was frequently described as co-occurring with male partner alcohol use. A majority of women referenced oral PrEP as their preferred method for HIV prevention, highlighting the agency and discretion it provided as beneficial attributes for women experiencing IPV. Fear of judgement from peers for remaining with an abusive partner and a lack of clear community messaging around IPV were identified as barriers to disclosure and support-seeking. Addressing the lack of social support received by women experiencing IPV during pregnancy in South Africa is essential to comprehensive IPV programming.

## Introduction

Gender-based violence refers to any violence perpetrated on the basis of gender. Intimate partner violence (IPV), the most prevalent form of gender-based violence, refers to any behavior towards an intimate partner that leads to harm, including emotional, verbal, physical and sexual violence and other controlling behaviors (e.g. restricting one’s movement and access to financial resources) [1]. Women in South Africa experience exceedingly high levels of IPV with lifetime prevalence estimates ranging from 20 to 50% [2–5]. IPV can adversely impact both mental and physical health: it can result in serious injury, and is associated with mental health disorders (depression, anxiety and post-traumatic stress disorder) as well as long-term health complications related to chronic exposure to violence and stress [6–9]. IPV is also a risk factor for HIV acquisition (through reduced ability to safely negotiate condom use and forced sex). Among persons living with HIV, IPV is associated with poor outcomes throughout the HIV continuum [10, 11]. In its most severe form, IPV can result in death. In South Africa, intimate femicide accounts for more than half of all female murders (56%) [12]. More than one in four women in South Africa experiences IPV during pregnancy (25-35%) [13, 14]. In addition to the aforementioned risks, pregnant women who experience IPV are more likely to have poor engagement and retention in antenatal care, and experience adverse birth outcomes as well as postpartum depression [7, 15, 16].

Patriarchal gender ideologies that uphold inequitable gender norms at both the dyad (intimate partnership) and societal level underpin the high rates of IPV observed in South Africa [17]. Extant research suggests that hallmarks of hegemonic masculinity specifically (e.g. exertion of force over intimate partners, use of violence, substance use) are frequently endorsed and males and females alike promote gender inequity and normalize physical and sexual violence against women in intimate partnerships [18, 19]. Contextually, much of this violence occurs while one or both partners are under the influence of alcohol. Alcohol use has also been identified as a means of coping with IPV victimization [20]. Pregnancy and parenthood represent a uniquely stressful and challenging time for couples, suggesting that women who experience IPV in pregnancy may experience additional risk factors for violence [21, 22]. Understanding patterns of IPV among pregnant women and identifying barriers and supports for reducing violence in this population is critical to the development of tailored effective evidence-based IPV prevention programming.

Experiences of IPV are also intertwined with the HIV epidemic in sub-Saharan Africa. HIV incidence and prevalence during the pregnancy and postpartum periods in South Africa are exceedingly high [23, 24]. Pregnant and postpartum women who experience incident HIV infection also experience higher rates of onward transmission due to the high maternal viral load during the acute phase of HIV infection [25], which can increase risk of vertical HIV transmission across the placental barrier or via breastmilk. While it is known that IPV is associated with HIV acquisition (e.g., through condomless sex and sexual violence) [10, 26–28], its impact on HIV pre-exposure prophylaxis (PrEP) use is understudied. Despite its tremendous promise, and recent guidelines that advocate for PrEP prescription and counseling for pregnant women not living with HIV in South Africa [29], continuation and adherence is low [30]. Understanding how IPV influences decision making around HIV prevention (including use of biomedical prevention) among pregnant women with characteristics associated with HIV acquisition is important for intervention planning. The present analysis qualitatively explores experiences of recent IPV among pregnant women residing near Cape Town, South Africa to identify potential points of intervention and understand how IPV may impact their approach and preferences towards HIV prevention, including oral PrEP use.

## Methods

### Participants

The present study was nested within Pre-Exposure Prophylaxis in Pregnant and Post-Partum Women (PrEP-PP) study (Clinical Trial Reg: NCT03902418, 10/01/2019). Methods of PrEP-PP have been described in detail previously [31]. In brief, pregnant women were recruited from antenatal care between August 2019 and October 2021; eligibility criteria included: 1) ≥ 16 years of age, 2) confirmed HIV-negative serostatus by a fourth-generation antigen/antibody combination HIV test, 3) confirmed pregnancy, 4) intention to remain in Cape Town through the postpartum period, and 5) no contraindications to oral PrEP. Study staff collected survey data and biological specimens at baseline and three-month intervals through postpartum. All participants were offered oral PrEP (TDF/FTC); initiation was not a requirement for participaton in the cohort.

We purposively sampled twenty participants from PrEP-PP to participate in in-depth interviews. Design and methods of this nested qualitative study have been described elsewhere [32]. Briefly, baseline data from PrEP-PP were used to identify participants with first-hand experiences of IPV and/or alcohol use coupled with PrEP use during pregnancy. Eligibility criteria included: (1) initiated PrEP at first ANC visit and (2) reported alcohol use during pregnancy and/or IPV in the past 12 months. Those selected and interested came to the clinic for further screening. Informed consent was obtained from all eligible subjects prior to participation in the in-depth interview. Ethical approval was obtained from the Human Research Ethics Committee at the University of Cape Town and the University of California, Los Angeles Institutional Review Board.

### Procedures

Interviews were collected between September and November of 2022 by a trained qualitative researcher. All women were postpartum at the time of their interview. Interviews were conducted in isiXhosa, the local language, and audio recorded. Topics included (1) drivers of IPV and perinatal alcohol use; (2) patterns of drinking among pregnant and postpartum women in South Africa; (3) decision making around HIV risk reduction; and (4) reasons for PrEP continuation and adherence. Findings around patterns and drivers of IPV and HIV risk and prevention are presented below. Given the sensitive nature of the topic and to minimize social distance between participant and researcher, the interviewer was a young South African woman who was also a mother herself.

Interviews were transcribed and translated into English by the interviewer and another South African female researcher. Data were then www.dedoose.com coded and analyzed in Dedoose (version 9.0.86) (www.dedoose.com) by two researchers, with parallel double coding of an initial batch of transcripts (*n* = 4, 25%) done to ensure consistent code application and iteratively refine the codebook. Both deductive and inductive methods were used for codebook development. After the fourth transcript was reconciled, the remaining transcripts were coded. To facilitate intercoder reliability, these remaining transcripts were independently coded by the same two researchers, in conjunction with meetings to facilitate reflexivity. The two primary analysts (APM and SSD are both US-based female public health researchers). Analysis activities were conducted by a diverse and multidisciplinary study team that was all female and predominately South African with extensive IPV and maternal health research experience in South Africa. A collaborative team-wide meeting was held to discuss emergent themes followed by subsequent rounds of data synthesis and discussion. Interpretive thematic analysis was performed to understand our results [33]. Team meetings emphasized reflexivity, with discussion of positionality, personal biases, and the wider social, political and historical context. Steps were taken throughout the study to improve the rigor of our work through application of the four criteria for establishing trustworthiness [34]: dependability (routine peer debriefs with the study team, audit trail), credibility (use of initial parallel coding to ensure code application consistency), confirmability (reflexivity) and transferability (purposive sampling, contextualization, thick description). Findings from this study were very rich, precluding inclusion of all results in a single manuscript. We separated our analysis into two separate manuscripts: the present paper and one focused on perceptions of alcohol use in pregnancy.

## Results

Interviews were conducted among twenty postpartum women, all of whom reported safe and successful deliveries (a requirement of continuation in the cohort postpartum) and all of whom reported taking oral PrEP during pregnancy (mean postpartum age = 30.25 years, SD = 6.16) and 10% (*n* = 2) of whom were still on PrEP at the time of their interview. The majority of participants (*n* = 16, 80%) reported experiences of IPV within the past 12 months, with four in ten women (*n* = 8, 40%) reporting past year alcohol use and 20% reporting both, according to data from their baseline study enrollment in PrEP-PP. However, it is worth noting that additional participants indicated experiences of recent IPV and/or alcohol use during pregnancy at the time of their postpartum interview. Results have been organized into the four most salient emerging themes: (1) patterns of IPV during pregnancy (2) barriers to seeking support among those with IPV experiences, (3) impact of IPV on HIV prevention decision making, (4) PrEP use in the context of IPV.

### Patterns of IPV during pregnancy and postpartum

Participants described experiences of verbal/emotional, physical, and sexual IPV during, before and after pregnancy. Some women described a change in their relationship dynamics (for better or worse) when they learned they were pregnant. A couple of participants described experiencing an increase in IPV (including some experiencing IPV for the first time after learning they were pregnant); one participant described an escalation of the IPV she experienced from verbal/emotional IPV prior to pregnancy to physical violence during pregnancy.

*The abuse started when I was pregnant. I do have a thought that says that he was abusing me before that, I just wasn’t paying any attention [to it though] and he wasn’t hitting me. He would say [abusive] words, but I wouldn’t pay attention, or I wouldn’t care…And then when he was being disrespectful, he would be disrespectful with his family. And then I thought, “you see here, I have no defender. I have no family in Cape Town. I came here to work, and I came to my friends. So let me try another way, but before that, let me wait for my heart to have enough.” (28 years old, reported no alcohol use during pregnancy and recent IPV)*.

Others reported continued IPV and conflict, such as the participant below, who described ongoing abuse during pregnancy which she felt was exacerbated by the effects of her pregnancy hormones:

*[My partner continued to hit me in pregnancy]… a pregnant person’s hormones… I will provoke them a lot more now, because I am pregnant, which is the very thing he may not like, the very thing that causes him to hit me. I am going to continue to say, because perhaps I have less self-control. I will say things like, “This is your habit, you want to kill this child, hit me.” You, see? If that’s something you did, you will do it even more when you are pregnant, because you can’t control yourself. Maybe you will even recall ways he upset you in this past. (28 years old, reported no alcohol use during pregnancy and recent IPV)*

For other participants, the abuse stopped during the period of pregnancy. For some, IPV resumed post-partum. In the instance below, the abuse then extended to the child:

He didn’t abuse me while I was pregnant, he started abusing me [again] after the child was born. He started abusing when the child was two or three weeks old. He [also] abused the child by taking the child while he was asleep and threatening to shred him to pieces with a saw and trying to throw him out. People had to come to the rescue, I screamed, and people came to the rescue. I told people that he was taking the child and threatening to cut him into pieces. (35 years old, reported no alcohol use during pregnancy (*but discussed drinking throughout her pregnancy when interviewed) and recent IPV)

Another participant described how her partner exerted control over her through verbal abuse and humiliation.

*He would say, “you are not fit to be a mother.” You see things like that. “I don’t know what kind of example you are going to be for my child.” And I would think to myself, “what have I done wrong?” This other time, like, I was wearing clothes, we were in the middle of a fight. He said, “take everything that you are wearing off, because it was bought with my money.” I thought he was joking. He said, “girl, I am saying take off my clothes that were bought with my money.” But then there was no one around. I am telling you, he made me undress and left me to be in my underwear. I was there, sitting. And then I tried to cover myself with a blanket, he said, “don’t cover yourself, stay right there, sit on that chair, just like that.” (35 years old, reported no alcohol use during pregnancy and recent IPV)*.

Alcohol use emerged as a catalyst for IPV in several narratives. Here one participant describes how she experienced IPV prior to learning she was pregnant and how this violence only happened in the context of alcohol use:

*He wouldn’t hit me randomly; he wouldn’t hit me when we were hanging out just like this. It was when we were drinking. We would drink together and then we would fight and hit each other. I also fought back, I wouldn’t sit back, I wouldn’t leave him. (23 years old, reported no alcohol use during pregnancy and recent IPV)*

### Experiences in seeking support among those experiencing IPV

Among those experiencing IPV, reluctance to disclose to friends and family was a recurring theme. There was a general perception that if one disclosed the IPV they experienced, friends or family would compel them to leave the relationship. If they stayed, they would be judged, a decision which carried stigma. This fostered a sense of privacy around relationship dynamics to avoid stigma and judgment from family and peers as described by the woman below.

*No, no I never told anyone [about the abuse]. My problems are my problems. I solve them how I solve them. The way they can be solved. And, people like to judge, a person may come and judge you because of your things, whilst maybe they may be experiencing the same problem or an even bigger problem. So, I chose to just keep quiet or solve my problems (22 years old, reported alcohol use during pregnancy and recent IPV)*.

Women’s experiences in receiving support from the general community around IPV were mixed. One woman described an instance of witnesses standing by while her partner beat her:

*You know, let me tell you, in my street, they don’t pay any mind to those things [violence], no ways. Like they don’t have time for that, because, my boyfriend once hit me. Like in the middle of the street and he chased me. He kicked me in my face, like in the street. Not a single person said anything, people were just standing in their yards. Just like that. (22 years old, reported alcohol use during pregnancy and recent IPV)*

Another described community members intervening to give her support after being hospitalized for abuse, explaining how she ultimately didn’t press charges against her abuser as she had no money and nowhere to go:

*[Community leaders] called the police the first time it happened. The police took him and he got arrested. And then on the second time, he was just starting to hit me in front of people, the community members hit him. And then third time, he hit me so hard that I fainted for three days, while my belly was this big. I came back, the doctor was going to have him arrested. And then they called a social worker for me, they said that I needed to wait for a Social Worker before I got be discharged. And then they would call the police. I said, “My doctor, I hear you. You want to have him arrested. I won’t be able to have him be arrested because I have no place to live. Because even now, where will I go after I get discharged from the hospital?” And then the doctor said, “What are you saying? What is your conclusion?” I said, “no, I am not going to have him arrested.” And I stayed. (28 years old, reported no alcohol use during pregnancy and recent IPV)*

The unpredictability of community response to IPV (apathy vs. intervention) may also influence women’s support seeking behavior. One participant described indifference when she tried to seek help at a local police station.

On this one occasion he took me out while his sister was present, he wanted to sleep with me, and I didn’t want to. He was forcing himself onto me. So, he hit the lamp…and then he said, “Open the door and leave.”…I left that house; he had a metal rod with him and he hit me with it. Even then, I tried to go to the police station. My hope was that they would take that metal rod away from him…When we got there they said that we were not under their district, we were under a different police station’s district and that we should go to that police. But then that police station was far, and it was at night. (35 years old, reported no alcohol use during pregnancy (*but discussed drinking throughout her pregnancy when interviewed) and recent IPV)

### Impact of IPV on ability to negotiate condom use

When asked how IPV impacted decision making around HIV prevention nearly all participants indicated it was a barrier. Participants described how fear of violence impacted their ability to safely negotiate condom use.

*I can say that it is not easy to ask for a condom when someone is abusive. It is not easy to ask for a condom when you are with someone like that. It is not easy. […] It is the same as when you are afraid of saying no to sex. For example, you may cross your legs and say, “no, I don’t know want to.” And he is going to grip you violently and say, “Why? Who slept with you?” You hear that. You eventually give in. And now do you think I can casually say, “argh, let me just go to the cupboard and grab a condom.”?(35 years old, reported no alcohol use during pregnancy and recent IPV)*.

In particular, fear of being accused of infidelity for asking to use a condom was mentioned as a barrier to use.

*Abuse impacts [condom use], especially for people like us, people who are married. Asking your husband to use a condom is very difficult, because he will say, “Why now? Do you have another partner? Is that why you’re asking us to use a condom?” (28 years old, reported no alcohol use during pregnancy and recent IPV)*.

### PrEP use in the context of IPV

In light of challenges related to condom use described above, many of the women indicated that PrEP was their preferred method of protecting themselves and their baby from HIV in the context of IPV. The most frequently cited reasons for initiating and adhering to PrEP were uncertainty and suspicions regarding their partner’s sexual behaviors outside of the partnership and wanting to protect the health of their unborn baby (through HIV prevention). Several women described how their partner was more likely to cheat while they were pregnant (in some cases this was suspected infidelity while in other cases it was confirmed), serving as additional motivation to initiate PrEP during this period.

*What made me decide to take PrEP is because I know that my partner really likes women a lot. I thought to myself, “You see, it’s going to become even worse when I am pregnant.” I had the thought that you don’t have a high sex drive when you are pregnant. Well, that’s my experience, let me say, I don’t have a high sex drive when I am pregnant. Perhaps it is different for others. Okay a person will think, “okay then let me go and cheat.” Even during the period when you are raising your child, men really like to cheat during that period, a lot of them. That’s why I thought that PrEP would work best for me. (35 years old, reported no alcohol use during pregnancy and recent IPV)*

This theme continued to present itself, even amongst participants without reported IPV experiences, highlighting the universal unpredictability of both partner sexual activity, unknown partner HIV serostatus as well as condom use during pregnancy.

*I was interested the moment they told me that PrEP protects from HIV. Because I know that I could sit and think that I don’t have another partner, but, I don’t know what my partner is doing. For example, we live far from each other now and they are saying that he has a girlfriend now. So, I said, “No, I want PrEP.” Because sometimes we don’t use a condom and sometimes, we do. So, I can say to myself, “I have one partner, all the while that partner is sleeping with everyone in the community.” (28 years old, reported alcohol use during pregnancy and no recent IPV)*.

PrEP was described by several participants as a way to take control of one’s sexual and reproductive health when their partner’s behaviors may increase their risk of incident HIV. One woman described how PrEP allowed her to avoid conflict that might otherwise arise regarding HIV prevention in her relationship.

*He didn’t want to use condoms when I suggested we use them. To the point where you are accused of cheating […] And then again, when you suggest going to get tested together the response is, “I’m not sick. Where is that coming from?” You are going to experience increased abuse when you talk about these things […] So that’s why I found PrEP to be safest way to protect yourself. Because you just take your pill, and you keep quiet. He would see the pills and I said to him, “these are PrEP pills, I am taking them to protect myself and the child from viruses.” (23 years old, reported no alcohol use during pregnancy and recent IPV)*.

Several women chronicled experiences of IPV as a barrier to optimal PrEP adherence. These reports included descriptions of violence related to PrEP use, with one account of a participants’ PrEP supply itself being destroyed by an intimate partner due to the misinterpretation of PrEP as antiretroviral therapy.

*The first day I took PrEP, I took it home and just put it there, alongside that piece of paper. There is a piece of paper that you get. I don’t know where I had left to, but the moment I got back he said, “All this time you have HIV?” Whack, whack, whack…. It turns out that he didn’t read the information sheet properly, he didn’t read the whole thing. I think he just saw the words, “when you have HIV.” He shouted, “here are the pills to prove it.” He took them and poured them into the bucket. (28 years old, reported no alcohol use during pregnancy and recent IPV)*

Another participant expressed anticipating accusations of infidelity related to PrEP use, citing that she refrained from disclosing her decision to initiate PrEP out of fear her partner would falsely speculate intentions of having multiple sexual partners.

*There are some people that you see and just think, “You see this one? If I tell him that I am HIV positive or for example, if I tell them that I am using PrEP. You can tell that he will question why you are using PrEP. He will assume that it means you are planning to cheat.” So that means that I couldn’t tell my boyfriend that I am using PrEP, because it is going to look like I am doing other things.… because I know him and he doesn’t live here, when I really think about it, it is just an assumption, because I don’t know how he would react, because I haven’t actually told him, but, to him, it is going to look like I am planning to cheat. (31 years old, reported no alcohol use during pregnancy and recent IPV)*

Still, for most women, PrEP was described as a useful and convenient HIV prevention strategy for someone experiencing IPV, especially among those that also engaged in heavy alcohol use, as described by one participant who highlighted its privacy and autonomy as providing peace of mind to those consuming alcohol.

*You see [PrEP], it is also good for those who drink. They can think, “Oh okay, I know that you don’t want a condom, or a condom is unavailable or putting on mine will take too long. Let me just take my pill.” And then relax. They can come with their forceful energy you know that you will be very relaxed. And then you wake up the next day and you take your PrEP. You don’t have to discuss anything with anyone, it is easy, you are relaxed. (35 years old, reported no alcohol use during pregnancy and recent IPV)*

Among women engaging in alcohol use, some noted how alcohol heightened their forgetfulness, muddling the typical orderliness of their PrEP routine. Others mentioned an aversion to mixing alcohol and medication, with experiences of delaying or intentionally skipping doses due to alcohol consumption varying by participant:

*Sometimes, I would be in the groove and not have time to drink it [PrEP]…or be drinking and just think to myself I don’t want to mix the medication and alcohol. (22 years old, reported no alcohol use during pregnancy and recent IPV)*

Overall, participants seemed aware of their risk of HIV infection and recognized barriers to utilizing traditional HIV prevention methods in their relationship. PrEP was viewed as a female centered way to protect themselves and their baby.

## Discussion

Our qualitative findings enhance the understanding of cultural, social, and individual factors shaping experiences of IPV among pregnant or postpartum women in Cape Town, South Africa. Descriptions of IPV among participants were diverse with respect to timing (occurring before, during or after pregnancy) as well as pattern (continued vs. discontinued during pregnancy and postpartum timeframes), emphasizing the heterogeneous nature of IPV events within this population. Participants highlighted how IPV experiences present challenges to using condoms as HIV prevention during this unique developmental period in the lifespan for women. Given South Africa’s staggering rates of IPV victimization among women combined with its high incidence of HIV, better understanding both the dynamics of IPV experiences as well as prevention preferences among pregnant or postpartum women can inform provider and policy approaches to supporting this population’s HIV prevention preferences.

Consistent with existing literature, many participants viewed IPV as consequential to their HIV prevention efforts, particularly regarding the safe negotiation of condom use with a partner [35]. While physical and sexual IPV are widely recognized as barriers to condom use regardless of pregnancy status, pregnant women face an additional challenge of convincing their partner(s) to use a condom under circumstances where there is no risk of pregnancy and therefore no need of pregnancy prevention [36]. This challenge, coupled with knowledge of unknown partner sexual activity and unknown partner HIV serostatus during the pregnancy and postpartum periods, was frequently tethered to participant explanations of oral PrEP preferred HIV prevention method (which women had control over) as compared to condom use which is dependent on the male partner’s agreement. PrEP attributes such as confidentiality (can be taken discreetly) and empowerment (through sexual health agency) were enthusiastically and explicitly noted by almost all postpartum women reporting experiences of IPV in our study.

Despite high acceptability, results from prior studies highlighting IPV’s influence on female PrEP engagement are mixed, suggesting IPV may act as both a facilitator and barrier to PrEP use among women [37–39]. While some study participants referenced PrEP as a tool which minimizes conflict, several others cited experiences or fears of violence negatively impacting their decisions or ability to use PrEP, supporting this incongruity. Relatedly, research among women living with HIV have thus far indicated that experiences of IPV may obstruct uptake, continuation, or adherence of ART [9, 10, 40]. Challenges related to PrEP use in this population, coupled with suboptimal adherence [30] and uncertainty around partner serostatus underscore the importance of engaging male partners of pregnant women in HIV testing services. Couples HIV testing, in this case, through antenatal care, offers an entry point for male partners to enter the test and treat cascade and should continue to be a priority in this setting.

Additional maternal incentives to prevent HIV acquisition during pregnancy or postpartum periods may serve as a key component in the relationship between experiences of IPV and PrEP engagement. Recent analysis from our parent study revealed alcohol use prior to pregnancy (hazardous or any) was associated with increased engagement in HIV risk behaviors as well as increased odds of early PrEP continuation and adherence among those initiating PrEP [30]. Our forthcoming companion qualitative manuscript corroborated that women who reported alcohol use in pregnancy found oral PrEP to be a more reliable method of HIV prevention than having to engage in preventative efforts in the moment (while under the influence of alcohol) [32]. Collectively, this body of work suggests that mothers are highly motivated to protect themselves and their child as well as keenly aware of cumulative and increased risk during pregnancy (e.g., limited capacity for consistent condom use or partner's unknown sexual activity), indicating that oral PrEP may better facilitate optimal HIV prevention in contrast to condom use in this population.

Many of the postpartum women in our study reported some level of alcohol use during pregnancy, some of which referenced partner or self-consumption of alcohol as influential to their HIV prevention program. Beyond co-existing as individual risk factors for HIV, evidence suggests alcohol use and IPV behave synergistically, further augmenting a woman’s vulnerability to HIV acquisition [41]. Findings from PrEP-PP [42] as well as a large body of extant literature [20, 43] suggest that alcohol use may be a coping mechanism as well as precursor to experiences of partner violence This knowledge may offer clarity regarding participant’s references of “planning ahead” as far as PrEP regimen in the context of alcohol consumption, underscoring the acceptability and compatibility of PrEP among women confronting multiple, overlapping HIV risk factors.

There have been great strides within South Africa over recent decades related to gender equity and empowerment attributable to legislation reform and community mobilization. IPV, however, remains one of South Africa’s public health priorities, as observed by its severe prevalence and burden of disease, second only to HIV [17, 44]. IPV sits adjacent to harmful gender and social norms, making disclosure of experiences challenging for many. And yet, a duality was described where violence was both normalized and tolerated on a societal level while women also felt the expectation was that they should actively remove themselves from a violent relationship if they recognized they were in one. This expectation places a tremendous burden on the victim; especially if they happen to be pregnant and economically dependent on their male partner. This was emphasized in our sample, as several women anticipated judgment from peers as well as a deficit in consistent and supportive community messaging around IPV. Unmet needs, limited access to resources and resulting health consequences observed among South African women underscore the urgency for implementation of universal IPV screening and referral for services, even among those not disclosing IPV. Evidence from the MTN-020 trial, conducted in four sub-Saharan African countries including South Africa, suggests that development of tailored standard operating procedures to improve training and service delivery around an IPV response in the context of PrEP delivery can benefit both providers and patients [45]. Furthermore, integration of such services into HIV counseling and testing (as a precursor to PrEP initiation) was found to be acceptable and feasible among multilevel stakeholders (including patients) in South Africa, an approach which would expand reach beyond those indicating an interest in PrEP [46]. Beyond screening, the development of comprehensive psychosocial or legal support programming, such as through peer navigation or mentorship, may be a most salient approach. Given the interrelated and bidirectional relationship between alcohol use, IPV and poor mental health, and the potential impact of these issues on PrEP initiation and adherence, screening for, counseling and referral to support services should be comprehensive, addressing all three health issues. Integration of such counseling may require provider training to support provider readiness and ensure counseling approaches are evidence-based. However, the evidence base on the impact of such IPV training programs on provider attitudes and screening behaviors have been inconclusive and comes entirely from high and middle income countries [47].

While addressing IPV as an epidemic requires broad and complex strategies, pregnant and postpartum women experiencing IPV may be an ideal population for PrEP intervention. Given the increased and regular engagement in the healthcare system during this period, antenatal settings offer an opportunity for safe and effective counseling regarding risk reduction and HIV prevention. In order to tailor PrEP options to the needs and preferences of pregnant women, it is imperative that this population be included in clinical trials of novel PrEP modalities [48]. For example, long-acting injectable cabotegravir (CAB-LA) may provide greater discretion in comparison to oral PrEP, thereby further reducing experiences of violence related to PrEP disclosure and HIV prevention [49, 50].

Our study has strengths and limitations. All interviews were conducted postpartum, which may have influenced participant recall and reporting. We theorize, however, that as the sample included only those with safe and successful birth outcomes, this temporal shift in perspective may have actually increased transparency of our findings. Secondly, due to our study’s qualitative form and primarily urban sample, findings may not necessarily apply to other pregnant and postpartum women across and beyond South Africa. However, our ability to identify and recruit women from the established PrEP-PP cohort, allowed for engagement of an incredibly vulnerable population. Building upon the existing trust and relationship these participants had with research staff, we were able to engage participants in conversation around highly sensitive and stigmatized topics.

## Conclusion

Findings illustrate the complexities regarding IPV dynamics (precursors, duration, intensity) among South African women during pregnancy and postpartum, emphasizing both the breadth of the issue as well as the need for tailored, multiprong solutions to address violence in this population. As evidenced by our study and a growing body of literature, experiences of IPV victimization often continue into pregnancy and postpartum periods, making this population a priority for both IPV and HIV prevention intervention. Comprehensive strategies acknowledging and engaging peer networks, as well as improved protocols specific to screening and referrals, may be pivotal in achieving necessary change. Descriptions of oral PrEP as a preferred HIV prevention method in the context of IPV victimization during pregnancy further emphasizes the need to ensure oral PrEP’s widespread accessibility as well as the inclusion of pregnant and postpartum women to emerging, long-acting HIV prevention modalities.

## Data Availability

The datasets used and/or analysed during the current study available from the corresponding author on reasonable request.

## References

[1] World Health O, Pan American Health O. Understanding and addressing violence against women: intimate partner violence. In. Geneva: World Health Organization; 2012.

[2] Gibbs A, Washington L, Willan S, Ntini N, Khumalo T, Mbatha N, Sikweyiya Y, Shai N, Chirwa E, Strauss M (2017). The stepping stones and creating futures intervention to prevent intimate partner violence and HIV-risk behaviours in Durban, South Africa: study protocol for a cluster randomized control trial, and baseline characteristics. BMC Public Health.

[3] Roman NV, Frantz JM (2013). The prevalence of intimate partner violence in the family: a systematic review of the implications for adolescents in Africa. Fam Pract.

[4] Shai NJ, Sikweyiya Y. Programmes for change: addressing sexual and intimate partner violence in South Africa. South Afr Crime Q 2015, 51(0).

[5] Mpondo F, Ruiter RAC, van den Borne B, Reddy PS (2019). Intimate Partner Violence and its Association with self-determination needs and gender-power constructs among rural South African women. J Interpers Violence.

[6] Sugg N (2015). Intimate partner violence: prevalence, health consequences, and intervention. Med Clin North Am.

[7] Alhusen JL, Ray E, Sharps P, Bullock L (2015). Intimate partner violence during pregnancy: maternal and neonatal outcomes. J Womens Health (Larchmt).

[8] Campbell JC (2002). Health consequences of intimate partner violence. Lancet.

[9] Yonga AM, Kiss L, Onarheim KH (2022). A systematic review of the effects of intimate partner violence on HIV-positive pregnant women in sub-saharan Africa. BMC Public Health.

[10] Hatcher AM, Smout EM, Turan JM, Christofides N, Stockl H (2015). Intimate partner violence and engagement in HIV care and treatment among women: a systematic review and meta-analysis. AIDS.

[11] Kuchukhidze S, Panagiotoglou D, Boily MC, Diabate S, Eaton JW, Mbofana F, Sardinha L, Schrubbe L, Stockl H, Wanyenze RK (2023). The effects of intimate partner violence on women’s risk of HIV acquisition and engagement in the HIV treatment and care cascade: a pooled analysis of nationally representative surveys in sub-saharan Africa. Lancet HIV.

[12] South African Department of Women Youth and Persons with Disabilities.: National Stratgic Plan on Gender-Based Violence & Femicide (GBVF): HumAN Dignity and Healing, Safety, Freedom & Equality in our lifetime. In. Pretoria, South Africa; 2020.

[13] Hoque ME, Hoque M, Kader SB (2015). Prevalence and experience of domestic violence among rural pregnant women in KwaZulu-Natal, South Africa. South Afr J Epidemiol Infect.

[14] Gass JD, Stein DJ, Williams DR, Seedat S (2010). Intimate partner violence, health behaviours, and chronic physical illness among South African women. S Afr Med J.

[15] Vintzileos A, Ananth CV, Smulian JC, Scorza WE, Knuppel RA (2002). The impact of prenatal care on postneonatal deaths in the presence and absence of antenatal high-risk conditions. Am J Obstet Gynecol.

[16] Tsai AC, Tomlinson M, Comulada WS, Rotheram-Borus MJ (2016). Intimate Partner Violence and Depression Symptom Severity among South African women during pregnancy and Postpartum: Population-based prospective cohort study. PLoS Med.

[17] Jewkes R, Morrell R (2010). Gender and sexuality: emerging perspectives from the heterosexual epidemic in South Africa and implications for HIV risk and prevention. J Int AIDS Soc.

[18] Harrison A, O’Sullivan LF, Hoffman S, Dolezal C, Morrell R (2006). Gender role and relationship norms among young adults in South Africa: measuring the context of masculinity and HIV risk. J Urban Health.

[19] Gottert A, Barrington C, McNaughton-Reyes HL, Maman S, MacPhail C, Lippman SA, Kahn K, Twine R, Pettifor A (2018). Gender norms, gender role Conflict/Stress and HIV Risk behaviors among men in Mpumalanga, South Africa. AIDS Behav.

[20] Backe EL, Bosire E, Mendenhall E (2022). Drinking too much, fighting too much: the dual disasters of intimate Partner Violence and Alcohol Use in South Africa. Violence against Women.

[21] Goodwin MM, Gazmararian JA, Johnson CH, Gilbert BC, Saltzman LE (2000). Pregnancy intendedness and physical abuse around the time of pregnancy: findings from the pregnancy risk assessment monitoring system, 1996–1997. PRAMS Working Group. Pregnancy risk Assessment Monitoring System. Matern Child Health J.

[22] Song-Choi PJ, Woodin EM (2021). Stress, attributions, and hostility as predictors of psychological intimate partner violence at the transition to parenthood. Psychol Violence.

[23] le Roux SM, Abrams EJ, Nguyen KK, Myer L (2019). HIV incidence during breastfeeding and mother-to-child transmission in Cape Town, South Africa. AIDS.

[24] Woldesenbet S, Kufa T, Lombard C, Manda S, Morof D, Cheyip M, Ayalew K, Puren A (2021). The prevalence of unintended pregnancy and its association with HIV status among pregnant women in South Africa, a national antenatal survey, 2019. Sci Rep.

[25] de Vincenzi I (2011). Triple antiretroviral compared with zidovudine and single-dose nevirapine prophylaxis during pregnancy and breastfeeding for prevention of mother-to-child transmission of HIV-1 (Kesho Bora study): a randomised controlled trial. Lancet Infect Dis.

[26] Campbell JC, Baty ML, Ghandour RM, Stockman JK, Francisco L, Wagman J (2008). The intersection of intimate partner violence against women and HIV/AIDS: a review. Int J Inj Contr Saf Promot.

[27] Maman S, Mbwambo JK, Hogan NM, Kilonzo GP, Campbell JC, Weiss E, Sweat MD (2002). HIV-positive women report more lifetime partner violence: findings from a voluntary counseling and testing clinic in Dar Es Salaam, Tanzania. Am J Public Health.

[28] Li Y, Marshall CM, Rees HC, Nunez A, Ezeanolue EE, Ehiri JE (2014). Intimate partner violence and HIV infection among women: a systematic review and meta-analysis. J Int AIDS Soc.

[29] Bekker LG, Brown B, Joseph-Davey D, Gill K, Moorhouse M, Delany-Moretlwe S, Myer L, Orrell C, Rebe K, Venter WDF (2020). Southern African guidelines on the safe, easy and effective use of pre-exposure prophylaxis: 2020. South Afr J HIV Med.

[30] Miller AP, Shoptaw S, Moucheraud C, Mvududu R, Essack Z, Gorbach PM, Myer L, Davey DLJ (2023). Recent Alcohol Use is Associated with increased pre-exposure Prophylaxis (PrEP) continuation and adherence among pregnant and Postpartum women in South Africa. J Acquir Immune Defic Syndr.

[31] Joseph Davey DL, Mvududu R, Mashele N, Lesosky M, Khadka N, Bekker LG, Gorbach P, Coates TJ, Myer L (2022). Early pre-exposure prophylaxis (PrEP) initiation and continuation among pregnant and postpartum women in antenatal care in Cape Town, South Africa. J Int AIDS Soc.

[32] Miller AP, Court L, Shoetz S, Knight L, Essack Z, Ntwasa C, Moopelo K, Wara N, Shoptaw S, Myer L et al. Qualitative study of experiences of alcohol use during pregnancy among women at risk of HIV in Cape Town, SA. *Social Science and Medicine - Qualitative Research in Health*. 2024. 10.1016/j.ssmqr.2024.100394 PMC13384464 42519503

[33] Braun V, Clarke V (2006). Using thematic analysis in psychology. Qualitative Res Psychol.

[34] Lincoln Y, Guba E (1985). Naturalistic Inquiry.

[35] Hatcher AM, Woollett N, Pallitto CC, Mokoatle K, Stockl H, MacPhail C, Delany-Moretlwe S, Garcia-Moreno C (2014). Bidirectional links between HIV and intimate partner violence in pregnancy: implications for prevention of mother-to-child transmission. J Int AIDS Soc.

[36] Ryan JH, Young A, Musara P, Reddy K, Macagna N, Guma V, Seyama L, Piper J, van der Straten A. the MTNMST: Sexual Attitudes, Beliefs, Practices, and HIV Risk During Pregnancy and Post-delivery: A Qualitative Study in Malawi, South Africa, Uganda, and Zimbabwe. *AIDS and Behavior* 2022, 26(3):996–1005. 10.1007/s10461-021-03454-y PMC8840901 34907478

[37] Stanton AM, O’Cleirigh C, Knight L, Davey DLJ, Myer L, Joska JA, Mayer KH, Bekker LG, Psaros C (2022). The importance of assessing and addressing mental health barriers to PrEP use during pregnancy and postpartum in sub-saharan Africa: state of the science and research priorities. J Int AIDS Soc.

[38] Roberts ST, Haberer J, Celum C, Mugo N, Ware NC, Cohen CR, Tappero JW, Kiarie J, Ronald A, Mujugira A (2016). Intimate Partner Violence and Adherence to HIV Pre-exposure Prophylaxis (PrEP) in African women in HIV Serodiscordant relationships: a prospective cohort study. J Acquir Immune Defic Syndr.

[39] Giovenco D, Pettifor A, Powers KA, Hightow-Weidman L, Pence BW, Celum C, Delany-Moretlwe S, Hosek S, Donnell D, Anderson PL (2022). Intimate partner violence and oral HIV pre-exposure prophylaxis adherence among young African women. AIDS.

[40] Mepham S, Zondi Z, Mbuyazi A, Mkhwanazi N, Newell ML (2011). Challenges in PMTCT antiretroviral adherence in northern KwaZulu-Natal, South Africa. AIDS Care.

[41] Russell BS, Eaton LA, Petersen-Williams P (2013). Intersecting epidemics among pregnant women: alcohol use, interpersonal violence, and HIV infection in South Africa. Curr HIV/AIDS Rep.

[42] Joseph Davey DL, Le Roux SM, Brittain K, Dovell K, Shoptaw S, Miller AP, Phillips TK, Zerbe A, Abrams EJ, Myer L (2022). Alcohol use and intimate partner violence in HIV-uninfected pregnant women in Cape Town, South Africa. AIDS Care.

[43] Shamu S, Abrahams N, Temmerman M, Musekiwa A, Zarowsky C (2011). A systematic review of African studies on intimate partner violence against pregnant women: prevalence and risk factors. PLoS ONE.

[44] Sere Y, Roman NV, Ruiter RAC (2021). Coping with the experiences of intimate Partner Violence among South African women: systematic review and Meta-synthesis. Front Psychiatry.

[45] Garcia M, Roberts ST, Mayo AJ, Scheckter R, Mansoor LE, Palanee-Phillips T, Reddy K, Naidoo Y, Akello CA, Gaffoor Z (2023). Integrating gender-based violence screening and support into the Research Clinic setting: experiences from an HIV Prevention open-label extension Trial in Sub-saharan Africa. AIDS Behav.

[46] Colombini M, Scorgie F, Stangl A, Harvey S, Ramskin L, Khoza N, Mashauri E, Baron D, Lees S, Kapiga S (2021). Exploring the feasibility and acceptability of integrating screening for gender-based violence into HIV counselling and testing for adolescent girls and young women in Tanzania and South Africa. BMC Public Health.

[47] Kalra N, Hooker L, Reisenhofer S, Di Tanna GL, Garcia-Moreno C (2021). Training healthcare providers to respond to intimate partner violence against women. Cochrane Database Syst Rev.

[48] Joseph Davey DL, Bekker LG, Bukusi EA, Chi BH, Delany-Moretlwe S, Goga A, Lyerly AD, Mgodi NM, Mugo N, Myer L (2022). Where are the pregnant and breastfeeding women in new pre-exposure prophylaxis trials? The imperative to overcome the evidence gap. Lancet HIV.

[49] Celum C, Grinsztejn B, Ngure K (2023). Preparing for long-acting PrEP delivery: building on lessons from oral PrEP. J Int AIDS Soc.

[50] Wara NJ, Mvududu R, Marwa MM, Gomez L, Mashele N, Orrell C, Moucheraud C, Kinuthia J, John-Stewart G, Myer L (2023). Preferences and acceptability for long-acting PrEP agents among pregnant and postpartum women with experience using daily oral PrEP in South Africa and Kenya. J Int AIDS Soc.

